# Insight into the shared pathogenic link between COVID-19 and pterygium: a systematic bioinformatics analysis with experimental validation

**DOI:** 10.1186/s41065-025-00500-w

**Published:** 2025-07-14

**Authors:** Tianyi Zhou, Xueyao Cai, Wenjun Shi, Xia Ding, Yuchen Cai

**Affiliations:** 1 Department of Ophthalmology, Shanghai Ninth People’s Hospital, Shanghai Jiao Tong University School of Medicine, 639 Zhi-Zao-Ju Road, Huangpu District, Shanghai, 200011 China; 2 Shanghai Key Laboratory of Orbital Diseases and Ocular Oncology, Shanghai, China; 3 Department of Plastic and Reconstructive Surgery, Shanghai Ninth People’s Hospital, Shanghai Jiao Tong University School of Medicine, 639 Zhi-Zao-Ju Road, Huangpu District, Shanghai, 200011 China

**Keywords:** COVID-19, SARS-CoV-2, Pterygium, Conjunctival fibroblasts

## Abstract

The outbreak of coronavirus disease 2019 (COVID-19) has resulted in a remarkable threat to global public health over the past few years. Despite the tremendous studies of COVID-19 ongoing, few have focused on the viral impact on the ocular surface. As one of the most common inflammatory diseases of the ocular surface, pterygium could be triggered under multiple environmental exposures. In the present work, we aimed at investigating the potential interactions between pterygium and COVID-19. Based on bioinformatic tools, we compared databases of COVID-19 and pterygium and screened for common differentially expressed genes (DEGs). Multifactor regulatory network and co-expression network of the common DEGs were analyzed. In vitro experiments, including siRNA knockdown using human conjunctival fibroblasts (HConFs) confirmed the bioinformatics results. Gene Ontology (GO) and Kyoto Encyclopedia of Genes and Genomes (KEGG) enrichment analysis implied that immune response was associated with COVID-19-induced ocular events. We then identified five common DEGs, including *ERP27, SYTL5, STXBP6, EXTL1* and *DIO2*, which was further validated by in vitro experiments. Three hub genes were further extracted which included *SYTL5, STXBP6* and *ERP27* through protein–protein interactions (PPI) network. Furthermore, we illustrated a regulatory network consisting of eight transcription factors (*STAT6B, GATA1, POU2F2, PGR, RBPJ, STAT3, CRTC1* and *HMGA1*) and one microRNA (*hsa-miR-384*). Overall, we investigated the common link between SARS-CoV-2 and pterygium in the modulation of gene profiles on the ocular surface. Our study proposed a novel insight into the common pathogenic mechanisms between COVID-19 and pterygium, which are associated with immune dysregulation and pathological proliferation, indicating a viral impact on pterygium susceptibility. This innovative perspective may enable a more comprehensive understanding and advance towards improved clinical prevention and treatment.

**Supplementary Information:**

The online version contains supplementary material available at 10.1186/s41065-025-00500-w.

## Introduction

The pandemic disease caused by severe acute respiratory syndrome coronavirus 2 (SARS-CoV-2), known as coronavirus disease 2019 (COVID-19), has triggered significant health and economic concerns globally since the first reported cases in December 2019 [1]. Rapid replication of SARS-CoV-2 in vivo may prompt a robust immune response, providing protection against infection. However, this immunopathological process could also trigger the onset of cytokine storm, which is responsible for acute respiratory distress syndrome (ARDS) and respiratory failure, considered the leading cause of death in COVID-19 patients [2]. In addition to pulmonary symptoms, ocular manifestations have been observed, including conjunctival hyperemia, foreign body sensation and tearing, along with viral detection in conjunctival swabs and tears [3]. Recently, the term ‘Long COVID’ has gained significant attention, a complex array of long-term complications affecting multiple organ systems after SARS-CoV-2 infection [4]. A minimum of 5% of infected individuals do not fully recover but rather develop chronic sequelae that substantially impede their physical and cognitive performance [5].

Pterygium is a chronic inflammatory disease of the ocular surface with a high prevalence around 12% globally [6]. It is often presented with dry eye, visual impairment and astigmatism. Characterized by wing-like conjunctival thickenings that migrate over the corneal limbus, the pathological overgrowth of the conjunctiva is usually accompanied with neovascularization and chronic inflammation [7]. Surgical excision is the major treatment strategy, but the risk of recurrence remains relatively high [8]. UV radiation is considered as the most significant risk factor, causing limbal barrier disruption for pterygium progression. Other causative factors include viral agents, genetic factors, immunologic and inflammatory processes [9].

Studies have demonstrated that SARS-CoV-2 necessitates angiotensin-converting enzyme 2 (ACE2) and transmembrane protease serine 2 (TMPRSS2) as its entry receptor [10]. It is noteworthy that both receptors are expressed in conjunctival and corneal epithelium [11], as well as in human primary pterygium cells and tissues [12], the ocular surface may represent a potential entry and transmission route for SARS-CoV-2. As COVID-19 infection activates multiple inflammatory pathways, which is also crucial to pterygium formation, uncovering the link between SARS-CoV-2 and pterygium may help better understand the viral impact on pterygium. Ocular complications after SARS-CoV-2 infection were not unusual [13], nevertheless, little case related to pterygium has been reported. Standing on clinical perspective, it is necessary to comprehend whether the pathological process of SARS-CoV-2 shared some similarities with pterygium, and whether it would induce or accelerate the progress of pterygium.

**Fig. 1:**
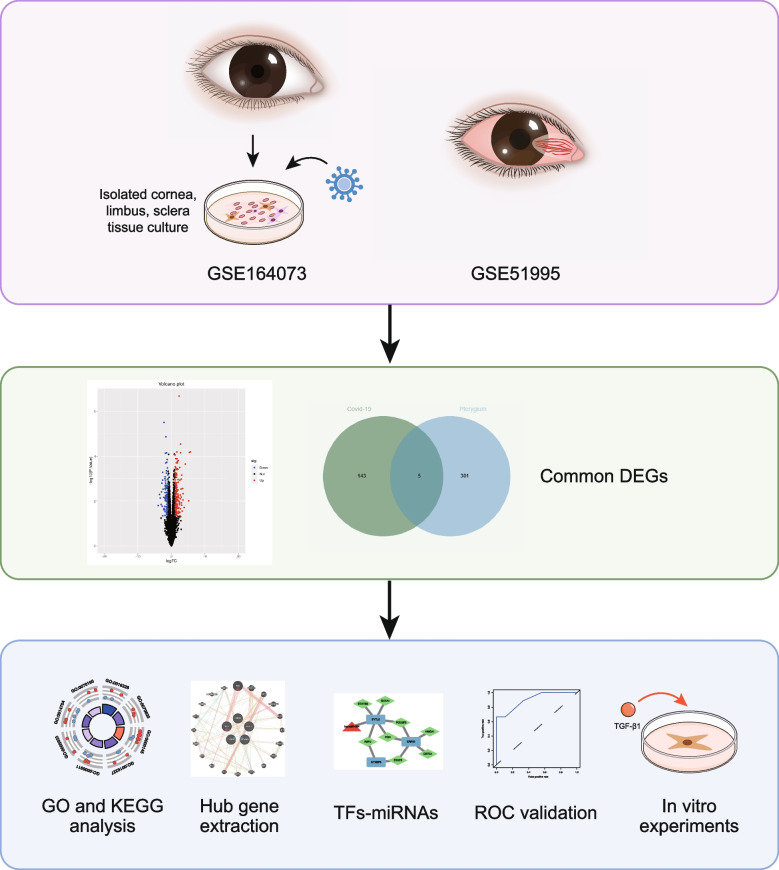
Schematic illustration of study. Differentially expressed genes (DEGs) were identified from two public datasets (GSE164073 and GSE51995). Subsequent functional enrichment analyses highlighted key biological processes and pathways involved. Hub genes were extracted using protein–protein interaction (PPI) networks. Regulatory elements including transcription factors (TFs) and microRNAs (miRNAs) were predicted, and hub genes were validated using receiver operating characteristic (ROC) curve analysis. In vitro experiments were performed to confirm gene expression changes for validation

Herein, through systematic bioinformatics, we aimed at investigating the potential interactions between pterygium and COVID-19. We first carried out functional enrichment analysis of differentially expressed genes (DEGs) in COVID-19 dataset to predict the functional alterations in SARS-CoV-2-infected ocular surface. Common DEGs between COVID-19 and pterygium were then screened, based upon which we constructed protein–protein interactions (PPI) and gene co-expression network. The interconnection of transcription factors (TFs) and microRNAs (miRNAs) related to the identified hub genes were outlined and validity of diagnostic efficacy was assessed by receiver operating characteristic (ROC) curve. As a final step, we conducted in vitro experiments to confirm the bioinformatics results. The methodology of the study was presented in a flowchart (Fig. 1).

## Material and methods

### Data source

The transcriptome data were obtained from the database Gene Expression Omnibus (GEO). The dataset GSE164073 contained gene expression profiling of SARS-CoV-2 infected cornea, limbus and sclera tissue isolated from human cadaver donors, compared with mock controls [14]. The dataset GSE51995 was selected as metadata set including four pterygium and four paired uninvolved conjunctiva samples [15]. GSE83627 consisting of endogenous gene expression differences between pterygium and conjunctiva tissues from four donors was appointed as the validation set. Gene count data were normalized with “edgeR” package.

### Functional enrichment analysis

Gene ontology (GO) analysis was carried out with the R"clusterProfiler"package, which includes categories such as biological processes (BP), cellular components (CC), and molecular functions (MF). Kyoto encyclopedia of genes and genomes (KEGG) enrichment analysis was conducted with the KOBAS database. The significant threshold was *p* < 0.001 and count ≥ 2. Furthermore, gene set enrichment analysis (GSEA) was carried out on the GSE51995 dataset to interpret the functional aspects of the pterygium data, utilizing GSEA software (version 4.1.0).

### Characterization of common DEGs between COVID-19 and pterygium

The common DEGs in the datasets GSE164073 and GSE51995 were screened with a criterion value set as false discovery rate (FDR)-adjusted *p*-value < 0.05 using the Benjamini–Hochberg correction. The volcano plots were generated by the"volcano3D"R package for DEG visualization. For the Venn diagram, the Jvenn jQuery Javascript library plugin was used. The differential expression levels of the DEGs within the two datasets were shown as boxplots with Prism (version 9.4.0).

### Construction of gene co-expression and PPI network

We uploaded the DEGs to the online database GeneMANIA, that is able to interpret gene files and predict gene interactions to visualize their co-interaction network. PPI network of the shared DEGs was constructed by uploading the genes to the Search Tool for the Retrieval of Interacting Genes (STRING) database to decipher potential protein–protein interactions. The interaction threshold was set at combined scores ≥ 0.4, and the resulting network was visualized using the open-source software, Cytoscape (version 3.9.0). Furthermore, a CytoHubba plugin was employed to identify and highlight hub genes based on their degree of connectivity within the network.

### Construction of TFs-miRNAs-mRNAs regulatory network

We identified potential TFs and miRNAs potentially related to the hub genes through a comprehensive search using the online databases miRTarBase, TargetScan, starBase for miRNAs, and Enrichr platform for TF identification. The regulatory network consisting of TFs, miRNAs, and mRNAs was then constructed and visualized using Cytoscape (version 3.8.2).

### ROC curve analyses of hub genes

We employed the'pROC'R package to generate the receiver operating characteristic (ROC) curve and the area under curve (AUC) was determined to assess the prediction ability of the identified hub genes on the diagnosis of COVID-19 and pterygium.

### Culture and treatment of human conjunctival fibroblasts

Human conjunctival fibroblasts (HConFs) were purchased from ScienCell Research Laboratories (Carlsbad, USA), cultured in a high-glucose DMEM medium supplemented with 10% fetal bovine serum (FBS) and 1% penicillin/streptomycin at 37 °C with 5% CO_2_, 95% air. To simulate pterygium phenotype, HConFs were cultured with 10 ng/ml transforming growth factor beta 1 (TGF-β1, PeproTech) for 24 h. HConFs were transfected with 100 nM STXBP6 siRNA in 6-well plates according to the protocol provided by the manufacturer (Genomeditech). Cells treated with non-targeting siRNA (siNC) at equivalent concentrations served as controls. The sequence of siSTXBP6 was as follows: 5'−GGCGAAUAUUUAACUUAUA(dT)(dT)−3'(F), 5'−UAUAAGUUAAAUAUUCGCC(dT)(dT)−3'(R).

### Quantitative reverse transcription polymerase chain reaction (qPCR)

Total RNA was isolated from different subsets of HConFs seeded on 6-well culture plate using TRIzol reagent (Invitrogen), then RNA concentration was determined using the Nano-Drop 2000 software (Thermo Fisher Scientific). Subsequently, 2000 ng of RNA were reverse transcribed into complementary DNA (cDNA) with PrimeScript RT Reagent Kit (Takara) according to the manufacturer’s protocol. Quantitative PCR (qPCR) detection was carried out with the Hieff UNICON SYBR Green Master Mix (Yeasen) on a Real-time PCR Detection System (Applied Biosystems). The relative mRNA expression of the target gene was reported as the fold change relative to the controls, normalized to the expression of the housekeeping gene, glyceraldehyde-3-phosphate dehydrogenase (GAPDH). The specific primer sequences (Tsingke Biotech) are included in Table S1.

### Statistical analysis

Data processing and statistical analyses were performed with R software v.4.1.2 and GraphPad Prism 9.4.0. The two-sample Wilcoxon test and t-test was used to evaluate the significant differences. Benjamini–Hochberg method was employed to calculate adjusted *p* value. *P* < 0.05 was considered statistically significant (*p < 0.05, **p < 0.01, ***p < 0.001, ****p < 0.0001).

## Results

### Functional alterations in SARS-CoV-2 infected ocular surface

**Fig. 2:**
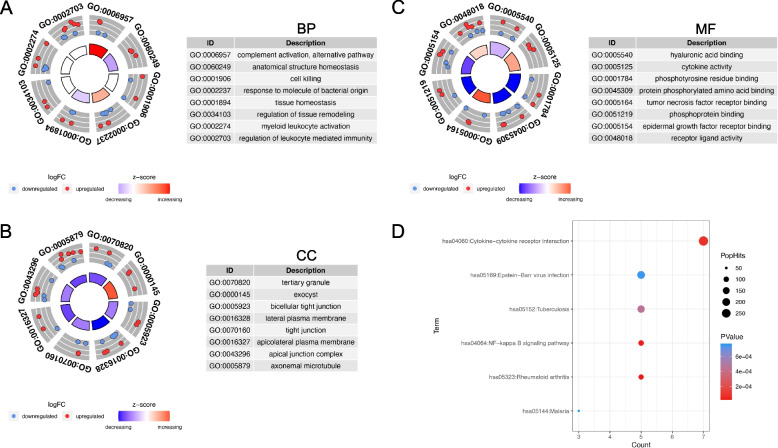
Functional alterations in SARS-CoV-2 infected ocular surface. **A**-**C** Gene ontology (GO) analysis of the differentially expressed genes (DEGs) in COVID-19 dataset, including (**A**) biological process (BP), **B** cellular component (CC), **C** molecular function (MF). **D** Kyoto Encyclopedia of Genes and Genomes (KEGG) functional enrichment analysis of the DEGs in COVID-19

**Fig. 3:**
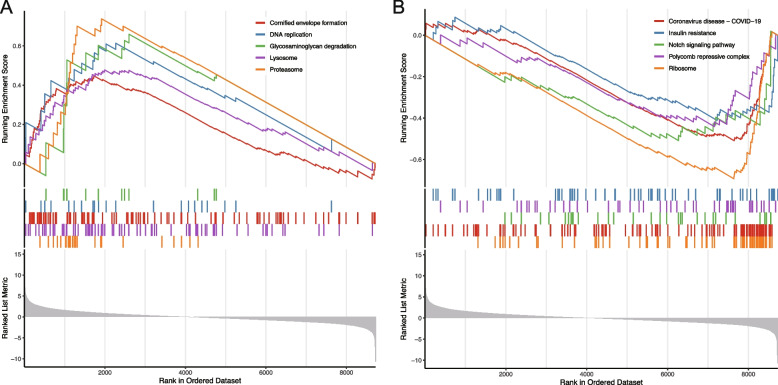
Gene set enrichment analysis (GSEA) based on the pterygium dataset GSE51995. **A** Top 5 upregulated pathways. **B** Top 5 downregulated pathways

We first performed GO and KEGG enrichment analysis of the DEGs in dataset GSE164073, in order to discover the molecular functions and pathways involved in ocular surface upon SARS-CoV-2 infection (Fig. 2 and Fig. 3). GO analysis revealed that the biological processes (BP) of the DEGs were mainly complement activation, alternative pathway, anatomical structure homeostasis, cell killing, response to molecule of bacterial origin and tissue homeostasis (Fig. 2A, Table 1). As for the cellular components (CC), we found that these DEGs were generally enriched in tertiary granule, exocyst, bicellular tight junction, lateral plasma membrane and tight junction (Fig. 2B, Table 2). Regarding to molecular functions (MF), it was shown in Fig. 2C that these genes mostly participate in hyaluronic acid binding, cytokine activity, phosphotyrosine residue binding, protein phosphorylated amino acid binding and tumor necrosis factor receptor binding (Table 3). KEGG analysis denoted that the DEGs were primarily involved in cytokine-cytokine receptor interaction, Epstein-Barr virus infection, tuberculosis, NF-kappa B signaling pathway, rheumatoid arthritis and malaria development (Fig. 2D, Table 4). In all, based on the functional enrichment analysis, we speculated that immune responses including cytokine receptor interaction and pathogen-activated pathways were mainly associated with SARS-CoV-2 infection on the ocular surface.

**Table 1:** BP of GO analysis of the DEGs in COVID-19 dataset

Term	Description	*P* value	Genes
GO:0006957	Complement activation, alternative pathway	9.80E-05	C3/CFB/C9
GO:0060249	Anatomical structure homeostasis	0.00029449	TNFAIP3/SLC39A8/TNFRSF11A/IL6/DCSTAMP/ILDR2/TSPOAP1/CLDN3
GO:0001906	Cell killing	0.00040591	C3/IL7R/STXBP2/C9/PTPN6/ICAM1
GO:0002237	Response to molecule of bacterial origin	0.00049462	TNFAIP3/DIO2/IRAK2/SOD2/TNFRSF11A/IL6/TLR2/IRF5
GO:0001894	Tissue homeostasis	0.0005056	TNFAIP3/SLC39A8/TNFRSF11A/IL6/DCSTAMP/ILDR2/CLDN3
GO:0034103	Regulation of tissue remodeling	0.00092345	TNFAIP3/TNFRSF11A/IL6/DCSTAMP
GO:0002274	Myeloid leukocyte activation	0.00132078	RELB/IL6/STXBP2/DCSTAMP/TLR2/PTPN6
GO:0002703	Regulation of leukocyte mediated immunity	0.00143841	C3/IL7R/IL6/STXBP2/PTPN6/ICAM1
GO:0001776	Leukocyte homeostasis	0.0016878	TNFAIP3/IL7R/IL6/TNFSF14
GO:0002673	Regulation of acute inflammatory response	0.00186046	C3/TNFRSF11A/IL6

*Abbreviations*: *BP* biological process, *GO* Gene Ontology, *DEGs* differentially expressed genes

**Table 2:** CC of GO analysis of the DEGs in COVID-19 dataset

Term	Description	*P* value	Genes
GO:0070820	Tertiary granule	0.00157632	TNFAIP6/STXBP2/FRMPD3/TMC6/PTPN6
GO:0000145	Exocyst	0.00298804	STXBP6/TNFAIP2
GO:0005923	Bicellular tight junction	0.00417562	ILDR2/CGNL1/CLDN7/CLDN3
GO:0016328	Lateral plasma membrane	0.00436175	SCN5A/CLDN7/CLDN3
GO:0070160	Tight junction	0.00478796	ILDR2/CGNL1/CLDN7/CLDN3
GO:0016327	Apicolateral plasma membrane	0.00514246	CLDN7/CLDN3
GO:0043296	Apical junction complex	0.00766062	ILDR2/CGNL1/CLDN7/CLDN3
GO:0005879	Axonemal microtubule	0.01546357	CFAP126/RIBC2
GO:0030666	Endocytic vesicle membrane	0.01783658	IL7R/STAB1/TLR2/WNT4
GO:0014704	Intercalated disc	0.02724573	DSG2/SCN5A

*Abbreviations*: *CC* cellular component, *GO* Gene Ontology, *DEGs* differentially expressed genes

**Table 3:** MF of GO analysis of the DEGs in COVID-19 dataset

Term	Description	*P* value	Genes
GO:0005540	Hyaluronic acid binding	0.00023307	TNFAIP6/ACAN/STAB1
GO:0005125	Cytokine activity	0.00127355	TNFSF15/IL6/TNFSF14/WNT8B/WNT4/IL32
GO:0001784	Phosphotyrosine residue binding	0.00161327	CBLC/PTPN6/GRAP
GO:0045309	Protein phosphorylated amino acid binding	0.00313939	CBLC/PTPN6/GRAP
GO:0005164	Tumor necrosis factor receptor binding	0.01064865	TNFSF15/TNFSF14
GO:0051219	Phosphoprotein binding	0.01129955	CBLC/PTPN6/GRAP
GO:0005154	Epidermal growth factor receptor binding	0.01201278	CBLC/GRAP
GO:0048018	Receptor ligand activity	0.01208905	TNFSF15/IL6/TNFSF14/ENHO/WNT8B/WNT4/IL32
GO:0030546	Signaling receptor activator activity	0.01298532	TNFSF15/IL6/TNFSF14/ENHO/WNT8B/WNT4/IL32
GO:0032813	Tumor necrosis factor receptor superfamily binding	0.02540041	TNFSF15/TNFSF14

*Abbreviations*: *MF* molecular function, *GO* Gene Ontology, *DEGs* differentially expressed genes

**Table 4:** KEGG analysis of the DEGs in COVID-19 dataset

Term	Description	*P* value	Genes
hsa04060	Cytokine-cytokine receptor interaction	8.54E-05	IL32, IL6, TNFSF14, TNFSF15, TNFRSF11A, IL7R
hsa05169	Epstein-Barr virus infection	0.00074609	IL6, TNFAIP3, RELB, ICAM1, TLR2
hsa05152	Tuberculosis	0.00044771	C3, IL6, CALML5, IRAK2, TLR2
hsa04064	NF-kappa B signaling pathway	3.22E-05	TNFSF14, TNFAIP3, TNFRSF11A, RELB, ICAM1
hsa05323	Rheumatoid arthritis	2.09E-05	IL6, TNFRSF11A, ATP6V1C2, ICAM1, TLR2
hsa05144	Malaria	0.00077009	IL6, ICAM1, TLR2

*Abbreviations*: *DEGs* differentially expressed genes, *KEGG* Kyoto Encyclopedia of Genes and Genomes

Additionally, we conducted GSEA in the pterygium dataset to further explore the molecular functions and pathways involved in the ocular surface. The top upregulated pathways identified include cornified envelope formation, DNA replication, glycosaminoglycan degradation, lysosomal function, and proteasome activity (Fig. 3A). Conversely, the top downregulated pathways encompass Coronavirus disease − COVID-19, insulin resistance, Notch signaling, the polycomb repressive complex, and ribosomal functions (Fig. 3B).

### Identification of the common DEGs between COVID-19 and pterygium

**Fig. 4:**
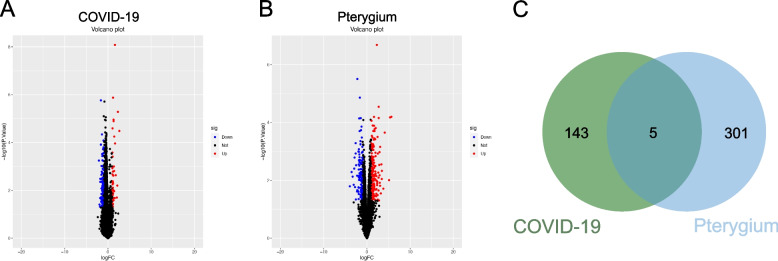
Identification of common DEGs from COVID-19 and pterygium dataset. **A** The volcano plot indicating 148 DEGs in the COVID-19 dataset GSE164073. **B** The volcano plot showing 306 DEGs in the pterygium dataset GSE51995. **C** The Venn diagram identified 5 common DEGs in the two datasets

Based on the dataset GSE164073 composed of genes expressed in SARS-CoV-2 infected human cornea, limbus and sclera tissues versus mock control, 148 DEGs were determined in total (Fig. 4A, top 50 DEGs listed in Table S2). The dataset GSE51995 contained pterygium and normal conjunctiva gene expression differences, through which we found 306 DEGs (Fig. 4B, top 50 DEGs listed in Table S2). Based on comparative analysis, 5 DEGs were spotted as commonly expressed (Fig. 4C).

### Analysis of the common DEGs

**Fig. 5:**
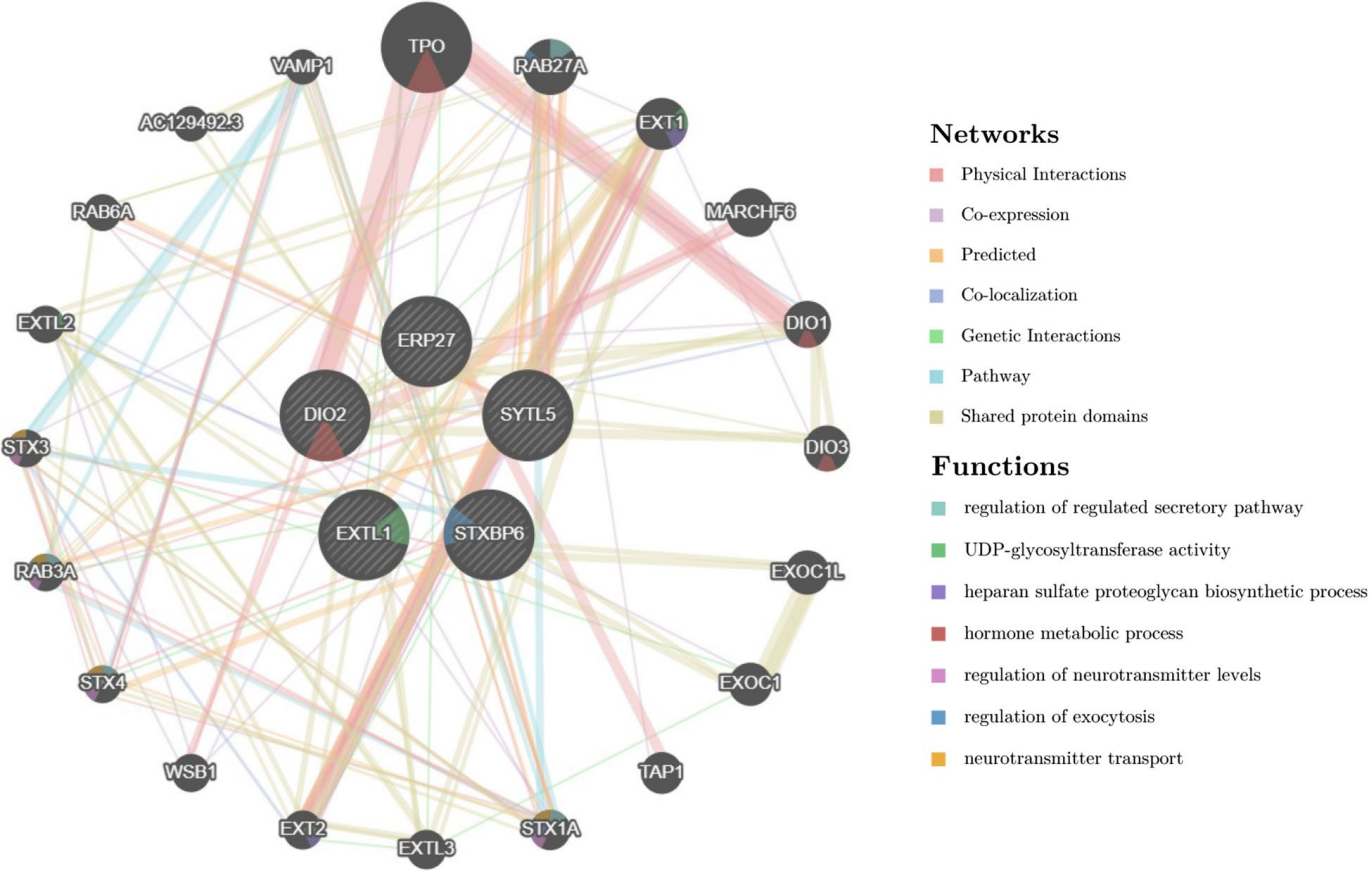
Common DEGs and their co-expression gene interaction network analyzed by GeneMANIA

The identified 5 common DEGs (*ERP27, SYTL5, STXBP6, EXTL1, DIO2*) were uploaded to the GeneMANIA database to investigate their interplay among physical interactions, co-expression, prediction, co-localization, genetic interaction, pathway and shared protein domains (Fig. 5). We also selected twenty genes in possible connection with the common DEGs. The gene interaction network demonstrated that these linked genes generally participated in the regulation of regulated secretory pathway, UDP-glycosyltransferase activity, heparan sulfate proteoglycan biosynthetic process, hormone metabolic process, regulation of neurotransmitter levels, regulation of exocytosis and neurotransmitter transport.

**Fig. 6:**
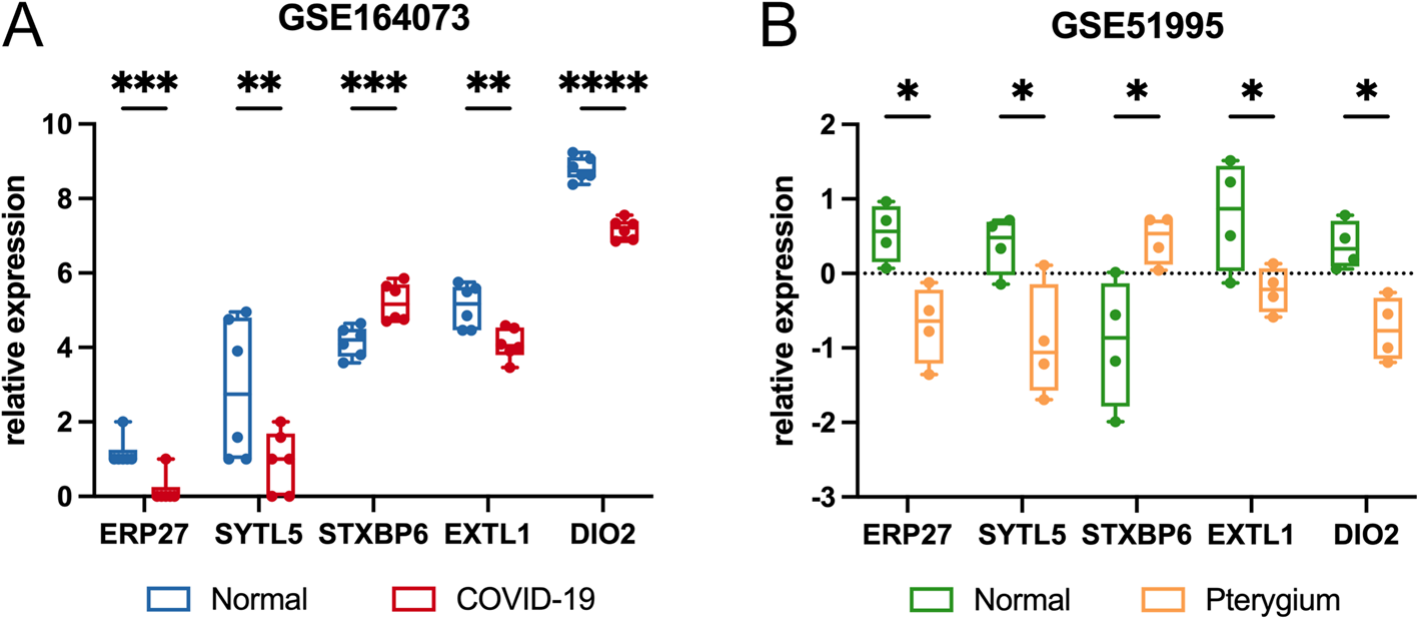
Differential expression level of the 5 common DEGs in (**A**) COVID-19 and (**B**) pterygium datasets. The vertical coordinates represent the relative gene expression levels. *p < 0.05; **p < 0.01; ***p < 0.001; ****p < 0.0001

The expression level of the common DEGs in the two datasets were then compared for validation (Fig. 6). Among the 5 common DEGs, four were significantly downregulated (*ERP27, SYTL5, EXTL1, DIO2*) and one was upregulated (*STXBP6*) in the COVID-19 dataset (Fig. 6A). A similar tendency was observed in pterygium tissues versus normal controls, with a significant decrease in the level of four genes (*ERP27, SYTL5, EXTL1, DIO2*) and an increment of gene *STXBP6* (Fig. 6B).

### PPI and TFs-miRNAs regulatory network

**Fig. 7:**
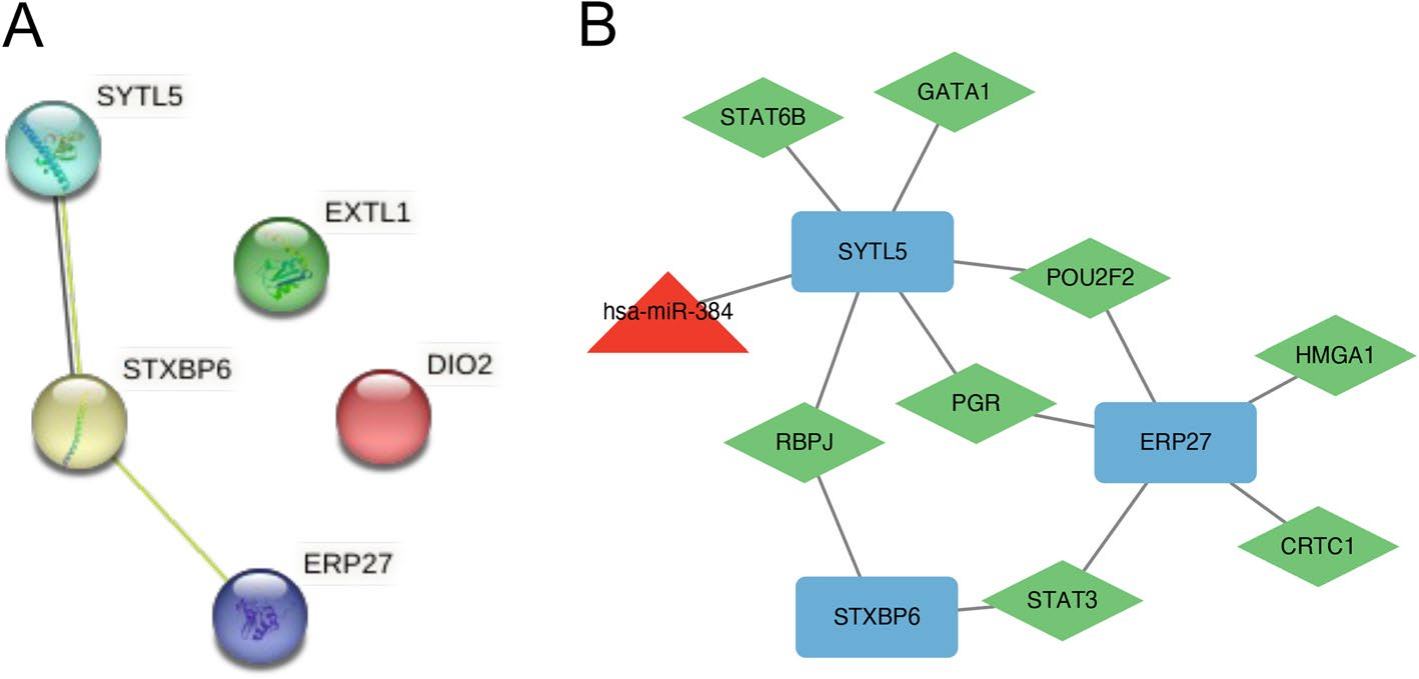
Characterization of the common DEGs in COVID-19 and pterygium. **A** Protein–protein interaction (PPI) network of the 5 common DEGs, three of which were extracted as hub genes. **B** Multifactor gene regulatory network incorporating the hub genes (mRNAs), microRNAs (miRNAs), and transcription factors (TFs). The blue rectangular nodes represent hub genes, the red triangular nodes represent miRNAs, and the green square nodes represent TFs

A PPI network was built from the identified common DEGs. Three hub genes were further extracted which included *SYTL5, STXBP6* and *ERP27* (Fig. 7A). Transcription factors (TFs) and miRNAs exert regulatory functions at distinct steps of gene expression. TFs mediate transcriptional regulation, while miRNAs predominantly act at the post-transcriptional level [16]. We screened for the potential TFs and miRNAs related to the 3 hub genes with online databases. The predicted TFs-miRNAs regulatory network included eight TFs (*STAT6B, GATA1, POU2F2, PGR, RBPJ, STAT3, CRTC1* and *HMGA1*) and one miRNA (*hsa-miR-384*) (Fig. 7B).

### Efficacy evaluation of the hub genes

**Fig. 8:**
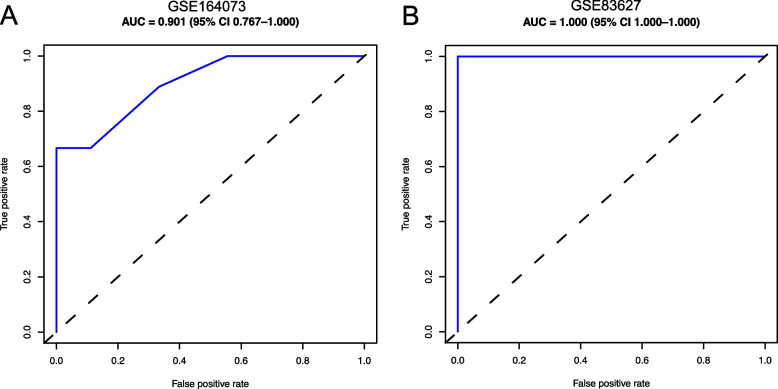
Receiver operating characteristic (ROC) curves for the three hub genes in (**A**) GSE164073 and (**B**) GSE83627. AUC, area under curve. CI, confidence interval

To evaluate the diagnostic efficacy of the 3 core genes, we performed the ROC analysis for the 3-gene signature within the datasets GSE164073 and GSE83627. As illustrated in Fig. 8, the combined gene signature showed an excellent diagnostic value for COVID-19 (AUC: 0.901) (Fig. 8A) and pterygium (AUC: 1.000) (Fig. 8B).

### In vitro validation of the common DEGs in HConFs

**Fig. 9:**
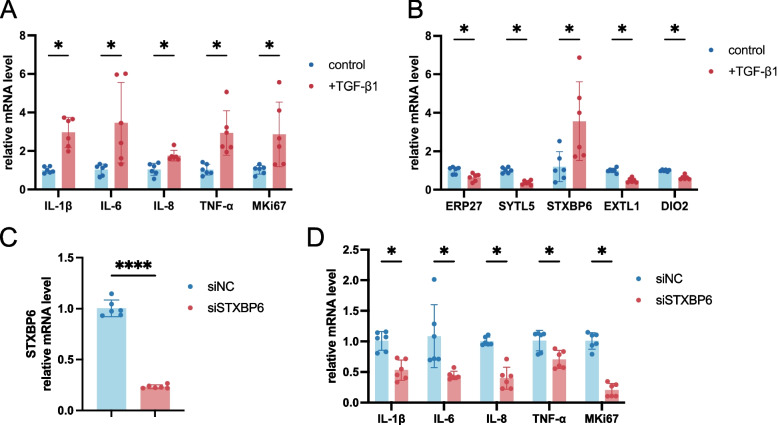
In vitro experiments using human conjunctival fibroblasts (HConFs). **A** Relative mRNA expression of proinflammatory markers IL-1β, IL-6, IL-8 and TNF-α, and proliferation marker MKi67 with or without TGF-β1 treatment. **B** Relative mRNA expression of the 5 DEGs (*ERP27, SYTL5, STXBP6, EXTL1, DIO2*) with or without stimulation of TGF-β1. **C** Relative mRNA expression of STXBP6 with siNC or siSTXBP6 treatment. **D** Relative mRNA expression of proinflammatory markers and proliferation marker with siNC or siSTXBP6 treatment. All data are represented as mean ± SD with dots stand for individual values. *P* values are expressed as *p < 0.05, ****p < 0.0001. TGF, transforming growth factor. TNF, tumor necrosis factor. IL, interleukin

Further validation of the DEGs was carried out using HConFs. As reported in various studies, pterygium tissues have been found to overexpress TGF-β1, an inducer of inflammatory infiltrates and proliferation [17]. We used TGF-β1 to simulate the cellular microenvironment of pterygium as previously described [18]. qPCR was performed to evaluate the mRNA expression levels of genes after TGF-β1 treatment. The increase of pro-inflammatory cytokines including IL-1β, IL-6, IL-8, and tumor necrosis factor alpha (TNF-α), as well as proliferation marker MKi67 was found in HConFs treated with TGF-β1 (Fig. 9A). Simultaneously, the expression levels of four DEGs were significantly downregulated (*ERP27, SYTL5, EXTL1, DIO2*), while *STXBP6* was upregulated after TGF-β1 induction (Fig. 9B).

Given that *STXBP6* was located at the core of the PPI network and was uniquely upregulated in both the pterygium and COVID-19 datasets, we conducted siRNA-mediated knockdown in HConFs to investigate its potential role. The knockdown efficiency of siSTXBP6 was confirmed by qPCR, demonstrating effective suppression of target gene expression (Fig. 9C). Notably, silencing *STXBP6* led to a significant reduction in the expression of proinflammatory cytokines as well as MKi67 (Fig. 9D). These findings suggest that *STXBP6* may contribute to conjunctival cell proliferation and inflammatory responses, both of which are pivotal processes in pterygium development.

## Discussion

It is believed that SARS-CoV2-mediated pathogenesis is primarily a result of immune evasion strategies and dysregulated cell responses, which boosts pro-inflammatory cytokines production. Ocular manifestations have been observed following SARS-CoV-2 infection or vaccination [19]. In view of the detection of ACE2 receptor on the ocular surface, it could be due to direct viral invasion or indirect immune dysregulation that caused the symptoms. Pterygium is a common ocular surface disease, though the exact mechanism undefined, its formation was associated with chronic inflammatory insults through epidemiologic studies [20]. This aroused our interest whether the hyperinflammation caused by SARS-CoV-2 would accelerate progression or the recurrence of pterygium. In order to find the crosslink over COVID-19 and pterygium, we used bioinformatic tools and analyzed online databases. Based upon the common DEGs explored, the coherent regulatory network was generated.

We identified 5 common DEGs (*ERP27, SYTL5, STXBP6, EXTL1, DIO2*) from the two databases GSE164073 and GSE51995. The differential expression levels of the five genes showed a similar trend in COVID-19 and pterygium datasets and in HConFs treated with TGF-β1, which to some extent validated their consistency in the development of diseases. Endoplasmic reticulum protein 27 (*ERP27*) is one of the member of PDI (protein disulfide-isomerase) family located in the endoplasmic reticulum it participated in the identification and catalysis of unfolded protein [21]. Studies suggest that expression level of *ERP27* may be associated with the outcomes in colorectal cancer and could potentially serve as specific predictive indicator for pancreatic cancer diagnosis and prognosis [22, 23]. Synaptotagmin-like 5 (*SYTL5*) was first characterized as a Rab27A effector protein involved in modulating cellular membrane trafficking events. Additionally, *SYTL5* was reported to promote the progression of papillary thyroid cancer through the nuclear factor-kappa B (NF-κB) signaling pathway [24]. Syntaxin binding protein 6 (*STXBP6*, also known as amisyn) was initially known as a partaker in SNARE (soluble N-ethylmaleimide-sensitive fusion protein attachment protein receptor) complex assembly which regulated exocytosis [25]. It was hypothesized to mediate the phagocytosis and antigen presentation of macrophages and monocytes as a part of immune response [26]. With its level of expression elevated in hepatocellular carcinoma, *STXBP6* was reported to have a regulatory role in the expression of PD-L1 in tumor cells [27]. Exostosin-like glycosyltransferase 1 (*EXTL1*), a member of the EXT family of tumor suppressor genes, was identified as a candidate neuroblastoma tumor-suppressor gene [28]. Required for the biosynthesis of heparan sulfate, *EXTL1* is involved in DC-mediated Th1 and Th17 development in the immune response against infection [29]. Iodothyronine deiodinases (DIOs) control the level of thyroid hormones, while DIO2 converts local T4 into its active form T3 and regulates intracellular T3 concentration [30]. It exerted an anti-inflammatory effect in chondrocytes through the production of T3 [31], yet others discovered that the loss of epigenetic silencing of *DIO2* could increase the risk of osteoarthritis [32]. Present studies suggested that Thr92Ala-DIO2 polymorphism was correlated with the mortality of patients with COVID-19 [33]. Taken together, we surmise that these core genes are in close association with the shared pathogenesis of pterygium and COVID-19. Although lacking direct evidence for now, they could be promising prognostic and therapeutic targets in the future.

The constructed TFs-miRNAs regulatory network constituted eight TFs (*STAT6B, GATA1, POU2F2, PGR, RBPJ, STAT3, CRTC1* and *HMGA1*) and one miRNA (*hsa-miR-384*). *STAT6B* and *STAT3* are two members of the STAT (signal transducer and activator of transcription) family that are involved in multiple biological functions. *STAT6B*, which is found to regulate the production of inflammatory cytokines upon LPS stimulation, has been suggested as a potential therapeutic target for VEGF-related diseases [34]. *STAT3*, on the other hand, has been extensively studied for its roles in regulating cell growth and immune function, as well as in the development of cancer and COVID-19 [35]. *GATA1* plays a crucial role in the differentiation, proliferation, and apoptosis of erythroid and megakaryocytic cells. Recent studies found that *GATA1* participates in the development of myeloid cells and modulates immune response [36]. *POU2F2* is a B-cell-specific transcription factor regulating B cell proliferation, differentiation, and the development of B-cell-derived tumors. It was reported to impact metastatic progression of gastric cancer and triple-negative breast cancer [37, 38]. Nuclear progestin receptor (*nPR* or *PGR*) is a progestin-activated transcription factor essential for ovulation, while the anti-inflammatory effects of progesterone have been considered beneficial in SARS-CoV-2 infection in cases of immune dysregulation [39]. *RBPJ*, a crucial TF in Notch signaling was found to promote glioblastoma progression through the IL-6/STAT3 axis in vitro [40]. *CRTC1* is a member of the CREB-regulated transcription coactivator (CRTC) family that plays a role in metabolism, aging, memory, and cancer [41]. The level of high mobility group A1 (*HMGA1*) increases especially in highly proliferative cells, possibly related to its regulatory role in tumor migration, invasion and stemness [42]. *MiR-384*, a central player in cancer cell proliferation, metastasis, and progression, also regulates Th17 cell polarization, which requires STAT3 activation [43, 44]. In light of these findings, the hub genes and TFs-miRNAs participate in diverse aspects of cellular physiological functions. Furthermore, they were found to regulate the pathological proliferation (carcinogenesis) and immune disorders. In some aspects, the development of pterygium is similar to that of tumors, such as fibrovascular proliferation and high recurrence rate [45, 46]. It is assumed that epithelial–mesenchymal transition (EMT) is responsible for pterygium recurrence [47], which is also an underlying mechanism of cancer metastasis. Meanwhile, immune infiltration plays critical roles in the progress of pterygium and COVID-19.

While our study offers new insights into the common genes associated with COVID-19 and pterygium, it is important to note that this study has several limitations. First, the transcriptome data used in this research were derived from a single COVID-19 and pterygium dataset with limited sample sizes, which may affect the statistical power and generalizability of our findings. Second, the transcriptome information in this study was collected from SARS-CoV-2-infected ocular surface tissue (cornea, limbus, sclera), while the pterygium dataset represents a part of the ocular surface. The data sources may not have been totally homogeneous, which might have contributed to variations in the transcriptome data. Third, while TGF-β1 stimulation was employed in vitro to mimic inflammatory and proliferative responses relevant to pterygium development, we acknowledge that it does not fully capture the immunopathological complexity of COVID-19. The use of pro-inflammatory cytokines such as IL-6 or IFN-γ might provide a more representative model of viral infection–induced inflammation. Future work would explore more refined conjunctiva-specific infection or immune-stimulation models to better replicate SARS-CoV-2–related ocular surface pathophysiology. Although we confirmed mRNA expression changes and related markers using qPCR, protein-level validation (e.g., via immunocytochemistry or Western blotting) was not performed in the current study. Future experiments will incorporate these approaches to further substantiate our findings. Additionally, the identified hub genes still require further in vivo validation to clarify their specific pathogenic mechanism.

In our study, we demonstrated that COVID-19 and pterygium share some molecular mechanisms during their progression on the ocular surface. In light of the viral damage to the ocular surface, especially immune system disorders, and the fact that pterygium is associated with disruption of the limbal barrier as well as inflammation, it might be possible that COVID-19 infection increases pterygium susceptibility. This study provides a new perspective on key biomarkers for the prevention and monitoring of pterygium, which can be explored in future studies. In addition, the results of our research may offer valuable insight for the advancement of research on the persistent manifestations of COVID-19.

## Conclusions

In the present study, we investigated the common link between SARS-CoV-2 and pterygium in the modulation of gene profiles on the ocular surface. Using bioinformatic methods we found five common DEGs (*ERP27, SYTL5, STXBP6, EXTL1, DIO2*), eight TFs (*STAT6B, GATA1, POU2F2, PGR, RBPJ, STAT3, CRTC1 and HMGA1*) and one miRNA (*hsa-miR-384*) that may participate collectively in the pathological process. Although further studies are required for direct validation, these novel genes could be potential targets for the prevention and treatment in COVID-19 and pterygium.

## Supplementary Information

Supplementary Material 1

## Data Availability

No datasets were generated during the current study.
