# Supplementary material for: Insight into the shared pathogenic link between COVID-19 and pterygium: a systematic bioinformatics analysis with experimental validation
Source: Hereditas. 2025 Jul 14;162:128. doi: 10.1186/s41065-025-00500-w (PMC12261744; doi:10.1186/s41065-025-00500-w)
Supplement: Supplementary file 1 — Supplementary Material 1 [file 41065_2025_500_MOESM1_ESM.docx]

Table S1. Primer sequences

| Gene | Forward primer | Reverse primer |
| --- | --- | --- |
| ERP27 | CCATACTCCATAGCATGGTGC | TGATGTTGTAGTGTGTCAGAACC |
| SYTL5 | ATGATCCTGGGCGTCCTAAAG | TCCCACTTCTACGTTTTGCTTC |
| STXBP6 | TTCTTGGCAACTGGAGGTCAA | TCGATACCATTAACCTGGCGAA |
| EXTL1 | GAAGATGCCGTTTCACCTCCT | CGCTGGGTACACGAATACCTT |
| DIO2 | TCCTCCTCGATGCCTACAAAC | GTGAGTAGACCAGTAGTCTGCT |
| IL-1β | ATGATGGCTTATTACAGTGGCAA | GTCGGAGATTCGTAGCTGGA |
| IL-6 | ACTCACCTCTTCAGAACGAATTG | CCATCTTTGGAAGGTTCAGGTTG |
| IL-8 | ACTGAGAGTGATTGAGAGTGGAC | AACCCTCTGCACCCAGTTTTC |
| TNF-α | GAGGCCAAGCCCTGGTATG | CGGGCCGATTGATCTCAGC |
| MKi67 | ACGCCTGGTTACTATCAAAAGG | CAGACCCATTTACTTGTGTTGGA |
| GAPDH | ACAACTTTGGTATCGTGGAAGG | GCCATCACGCCACAGTTTC |

Table S2. Top 50 DEGs for GSE51995 and GSE164073 based on *p* value

| GSE51995 | GSE164073 |
| --- | --- |
| ASPN+A2:A49, MLXIPL, MAMDC2, INHBA, KRT6C, TOP2A, GRP, CST6, CA3, CDH2, TYMS, MEOX1, KIF23, SLC16A2, CHST11, FAM49A, CPVL, BUB1, PLK2, MMP3, KIF4A, C20orf103, FOXM1, C2orf88, CPA4, BDKRB2, ECM1, BAMBI, NPNT, EMILIN2, IFNA2, CAPN6, ORC1, GREB1L, ASPM, GDNF, TMEM132A, VAV3, ATP6V0A4, POLE2, CD109, CDH4, ACTN1, KRT15, SH2D1B, CKAP2L, TNNI2, GPNMB, SCN7A, CREG2 | TNFAIP3, DIO2, MIR3142HG, RELB, TNFAIP6, IRAK2, SOD2, NOG, SLC39A8, SLAMF9, C3, GPRC5A, IL7R, DQX1, POU2F2, SMAD9, SLCO4A1, TNFRSF11A, ST8SIA1, LDHC, ACAN, DSG2, THSD7A, TNFSF15, STAB1, ERP27, IL6, KCNN3, MED4-AS1, STXBP2, DCSTAMP, HIST1H4C, STXBP6, LLGL2, ELOVL2, TP53AIP1, ERICH6, ILDR2, EPHX3, FAAH2, TSPOAP1, CBLC, CGNL1, FRMPD3, RIC3, PAPPA-AS1, KIAA1211, GS1-24F4.2, TNFAIP2, CFAP126 |
